# Bacterial genome reductions: Tools, applications, and challenges

**DOI:** 10.3389/fgeed.2022.957289

**Published:** 2022-08-31

**Authors:** Nicole LeBlanc, Trevor C. Charles

**Affiliations:** ^1^ Department of Biology, University of Waterloo, Waterloo, ON, Canada; ^2^ Metagenom Bio Life Science Inc., Waterloo, ON, Canada

**Keywords:** synthetic biology, bacteria, genome reduction, genome engineering, minimal genome

## Abstract

Bacterial cells are widely used to produce value-added products due to their versatility, ease of manipulation, and the abundance of genome engineering tools. However, the efficiency of producing these desired biomolecules is often hindered by the cells’ own metabolism, genetic instability, and the toxicity of the product. To overcome these challenges, genome reductions have been performed, making strains with the potential of serving as chassis for downstream applications. Here we review the current technologies that enable the design and construction of such reduced-genome bacteria as well as the challenges that limit their assembly and applicability. While genomic reductions have shown improvement of many cellular characteristics, a major challenge still exists in constructing these cells efficiently and rapidly. Computational tools have been created in attempts at minimizing the time needed to design these organisms, but gaps still exist in modelling these reductions *in silico*. Genomic reductions are a promising avenue for improving the production of value-added products, constructing chassis cells, and for uncovering cellular function but are currently limited by their time-consuming construction methods. With improvements to and the creation of novel genome editing tools and *in silico* models, these approaches could be combined to expedite this process and create more streamlined and efficient cell factories.

## 1 Introduction

From researching disease to producing various materials applicable in countless industries including food, pharmaceuticals, and textiles, bacteria have expanded what is possible and contributed to incredible advancements. This is highlighted by the industrial use of bacteria that naturally produce value added products such as antibiotics, amino acids, therapeutic products, biofuels, and materials for textiles and medical devices to name a few (Quillaguamán et al., 2005; Wendisch et al., 2006; Olano et al., 2008; De Eugenio et al., 2010; Choi and Lee, 2013; Shi et al., 2014; Jiang et al., 2018; Samrot et al., 2021). However, these processes rely, for the most part, on organisms that evolved in nature, and were not created for these industrial processes. Consequently, they have many cellular functions that are irrelevant to the desired application which limits the efficiency of producing the target end-product (Choe et al., 2016; Weiser et al., 2019). Breakthroughs in DNA synthesis and sequencing technologies and the ever-increasing data on metabolic pathways have enabled the creation of novel methods to engineer optimized bacteria with more efficient and higher yielding production in a multitude of applications.

Some of the greatest challenges within a cell that limit its applicability are complexity and absence of predictability. All bacterial cells are composed of a range and diversity of molecules interacting in complex networks for countless cellular functions (Fehér et al., 2007). This is further complicated by random mutations, some of them caused by mobile elements, that lead to unpredictable cellular behavior. To combat these issues, genomic reductions have been performed to remove dispensable genes from the genome. Genome sequencing and functional assays have revealed essential genes like those responsible for core survival, those involved in industrially relevant processes, and non-essential genes that contribute to genome instability and superfluous or unknown cellular functions (Fehér et al., 2007; Park et al., 2014). When using a cell to produce a specific biomolecule in a defined environment, many genes whose functions do not contribute to the intended process could be candidates for removal. This would allow for the creation of tailored cell factories with improved physiological characteristics for the specific application. This can also be more generalized to create a chassis with only the genes required for cell survival and proliferent growth that can be further engineered for downstream applications.

## 2 Benefits of a reduced genome cell

Prior to the publication of the first full bacterial genome, genomic reductions in *E. coli* were suggested based on the presence of genes unnecessary for growth under defined conditions (Koob et al., 1994). Further, the use of *Mycoplasma* strains as a model for minimal genome construction was suggested due to their naturally minimized genomes (Morowitz, 1984). Now, with countless constructed reduced genome strains, the benefits of these smaller genomes are evident. First, decreasing the number of genes and functions within a cell reduces the complexity of the organism and makes modelling of its metabolism and functional predictions much simpler (Choe et al., 2016). Next, genomic stability has been greatly improved in genome reduced strains. This is highlighted by the improved growth characteristics including genomic stability following the deletion of biosynthetic clusters in *Streptomyces chattanoogensis* (Bu et al., 2019). Also in *E. coli*, the deletion of error-prone DNA polymerases, that are expressed during SOS response and implicated in induced mutagenesis, resulted in a 50% decrease in the spontaneous mutation rate and improved genetic stability (Csörgo et al., 2012). Another advantage is the possibility of cells requiring less energy to replicate the smaller genome as well as lower transcriptional and translational costs. This correlation has not been fully investigated but many reduced genome strains display faster growth rates and higher cell density that could be attributed to these factors (Kolisnychenko et al., 2002; Mizoguchi et al., 2007; Zhu et al., 2017; Qiao et al., 2022). For example, the genome of *Lactococcus lactis* N8 was reduced by 6.9% by deleting prophages and genomic islands resulting in a shortened generation time by 17% (Qiao et al., 2022). Other observed benefits to genome-reduced strains include increased production of desired products, and improved transformation efficiency (Bu et al., 2019). Finally, the ease of genetic manipulation is one of the biggest advantages of a reduced genome strain. With improved growth characteristics, simpler metabolism, and fewer functions being performed within the cell, there is the potential to use it for many downstream applications such as expressing heterologous genes and producing biomolecules using tailored metabolic pathways.

## 3 Genome reductions occurring in nature

The idea of reducing bacterial genomes to improve physiological characteristics and create optimized hosts stems from this process occurring naturally through evolution. Cells evolve under strong selective pressure to maintain homeostasis against environmental changes. This forces cells to expand metabolic and signalling pathway redundancy to become more robust, which has resulted in increased genome sizes (Kurasawa et al., 2020). Thus, when cells are grown in laboratory and controlled conditions, this redundancy becomes unnecessary, making it possible to significantly reduce the size of the genome. This is displayed in obligately symbiotic bacteria that have undergone significant genome reduction through evolution (Choe et al., 2016). For example, *Buchnera* sp., an insect endosymbiont and a relative of *E. coli* (4.5 Mb genome), have genomes that are as small as 450 kb (Wernegreen, 2002; Fehér et al., 2007), approximately a 10th of the size of their *E. coli* relatives. Similar reductions have been observed in other obligate symbiont bacterial classes including member of the Gammaproteobacteria (*Buchnera aphidicola, Wigglesworthia*, *Blochmannia*) and Spirochaetes (*Borrelia burgdorferi*) (Fraser et al., 1995; Andersson et al., 1998). In these obligate symbionts, smaller genomes are possible because these bacteria have access to functions and metabolites from their host, obviating the need to encode them in their own genomes, compared to their free-living bacterial relatives. When a free-living bacterium becomes restricted to a host, the process of evolutionary genomic reductions begins with large and small deletions of genes no longer required, often accompanied by chromosomal rearrangements (McCutcheon and Moran, 2012; Bobay and Ochman, 2017). This further develops with long-term obligate symbionts of pathogens which lose many pseudogenes and almost all mobile elements, resulting in a more stable chromosome (Bobay and Ochman, 2017). To reach those tiny-genome symbionts, there is an ongoing gene loss as the organism evolves to survive in the given conditions (Bobay and Ochman, 2017). Compared to the smallest free-living bacteria, *Mycoplasma genetalium* with a genome of 580 kb, genome-reduced symbiotic bacteria have genomes two to four times smaller such as *Candidatus Tremblaya princeps,* a betaproteobacteria with a genome of 138 kb, *Candidatus Sulcia muelleri*, a bacteroidetes with a genome of 245 kb, and *Candidatus Hodgkinia cicadicola*, an alphaproteobacteria with a genome of 143 kb (McCutcheon and Moran, 2012).

While genomic reductions were first observed in obligately symbiotic bacteria, studies comparing genomic data reveal that they are also prevalent in free-living bacterial genomes (Wolf and Koonin, 2013; Albalat and Canestro, 2016). Genome evolution occurs both through expansion by horizontal gene transfer and duplication events, and through genomic reduction from large-scale gene deletion (Thomas and Nielsen, 2005; Lynch, 2006). Interestingly, bacterial genomes have a bias towards deletion events over expansion events and these large-scale deletions can occur in a relatively short evolutionary time frame, as highlighted by experimental evolution studies (Mira et al., 2001; Kunin and Ouzounis, 2003; Nilsson et al., 2005; Koskiniemi et al., 2012; Lee and Marx, 2012). Another important aspect to consider is the deleterious effect large-scale genomic reductions can have on a cell and how the organization of the genome needs to protect against this (Fehér et al., 2007). By computationally analysing the metabolism of 55 bacterial species at the genome scale, it was elucidated that the bacterial genomes are organized in such a way as to increase robustness of metabolic genes against the deletion of contiguous genes (Hosseini and Wagner, 2018). This is the result of segregation of essential and non-essential metabolic gene clusters and the separation of synthetic lethal gene pairs. The adaptive forces that favor this organization despite genomic elements like transposons that cause random genome rearrangements have been identified by computationally modelling a reduced bacterial cell. (Hosseini and Wagner, 2018).

## 4 Designing a reduced-genome bacterium

### 4.1 Determining gene essentiality

Prior to reducing an organism’s genome, studies are often performed to determine which genes are essential. Essentiality is entirely dependent on the environment the cells are grown in and their desired application. Genes that would be considered essential when grown on minimal media with limited nutrient supplementation, such as amino acid synthesis genes, would not necessarily be essential when grown on nutrient-rich complex media (Baba et al., 2006; Patrick et al., 2007). Frequently, there are already target genes that can be identified for deletion, such as genes in competing metabolic pathways when attempting to produce a specific biomolecule. But when looking to make significant genomic reductions, it is important to evaluate what genes are needed for survival in the given conditions. While the focus of this review is the application of gene essentiality determination studies in constructing reduced-genome strains, there are other applications of this data that are of value to note. Identifying genes essential to an organism’s survival will advance overall understanding of the fundamental principles of life (Moya et al., 2009; Jewett and Forster, 2010; Juhas et al., 2011; Commichau et al., 2013). Also, genes that are essential to a bacterium’s survival could be targets for novel antimicrobial development (Bumann, 2008; Juhas et al., 2012a, 2012b). Three approaches are commonly taken for determining gene essentiality in bacteria: comparative genomics, large-scale gene inactivation studies, and *in silico* modelling. All three of these routes have various advantages and disadvantages to the genome design and determination of essential genes often involves combining data from each approach.

#### 4.1.1 Essentiality determination by comparative genomics

First, the comparative genomics approach compares the genome of the target species to the genome of both closely and distantly related organisms to determine a core set of essential genes. Comparing the genomes of distantly related bacteria, 262 genes were found shared amongst them (Mushegian and Koonin, 1996). When more species were added into this comparison, the set of genes shrinks to 63 common core genes, mostly all related to basic components for gene expression and replication, identified across the Bacteria and Eukarya domains (Koonin, 2003). This can give a good idea of genes necessary for life across all species, but these alone would not be sufficient for survival. Accessory genes and metabolic genes are also required for survival in the given environment (Tarnopol et al., 2019). Additionally, proteins with similar functions may not necessarily have sequence similarities (Riley and Serres, 2000). Thus, the size of the minimal gene set may be underestimated as only those shown to be conserved across all species tested will be considered ‘essential’ and this also doesn’t include environmental dependence of genes (Fehér et al., 2007).

#### 4.1.2 Essentiality determination by gene inactivation

**FIGURE 1:**
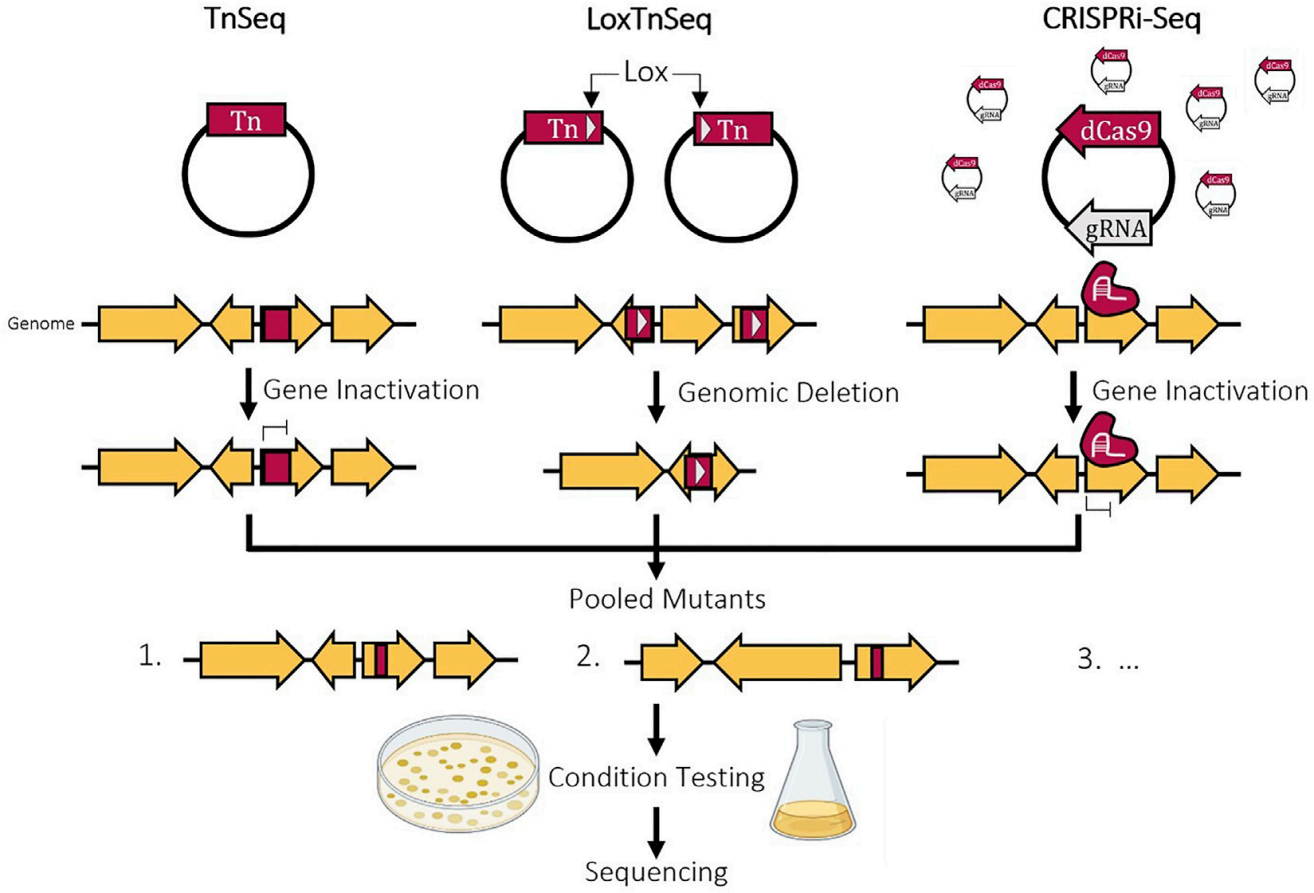
Schematic diagram of the construction of a reduced genome cell. Identification of essential genes using experimental and computational methods, genomic reduction by a top-down gene deletion or bottom-up synthesis approach, and evaluation of modifications of this strain.

Large-scale gene inactivation studies, such as transposon mutagenesis, can be utilized. These types of analysis are able to score mutants on their ability to survive. With technological advancements, the set of known essential genes is always evolving. This is highlighted by *B. subtilis* initially having a set of 271 ORFs considered essential in 2003 and reduced even further to 253 in 2014 (Kobayashi et al., 2003; Juhas et al., 2014). Transposon sequencing (TraDIS, Inseq, TnSeq) is a very common tool to use to identify essential gene regions in the genome and has been applied in many different species and for many different growth conditions (Goodman et al., 2009; Langridge et al., 2009; Wong et al., 2016; Dejesus et al., 2017; Higgins et al., 2017; Baby et al., 2018; de Maat et al., 2020; Johnson et al., 2020; Matern et al., 2020). This involves the creation of a saturated transposon library where each genomic region is interrupted and theoretically inactivated by a transposon (Figure 1). By culturing this library in different growth conditions, the cells that contain a transposon insertion in an essential genomic region will not survive. The location of the remaining transposons is determined using sequencing, identifying the genes that are nonessential. This can then be taken one step further by assigning a fitness score based on the transposon insertion frequency in every gene. This helps elucidate non-essential genes and quasi-essential genes that contribute to cell growth but are not essential to survival. While this method can rapidly identify thousands of nonessential genes across the entire genome, it only shows the effect of single mutations and gene knockouts on viability. This fails to capture epistatic interactions in the genome when multiple deletions are made such as synthetic lethal pairs or deletion combinations that could hinder the ability of the cell to grow rapidly (Yu et al., 2002, 2006). Also, the loss of individual genes can affect or control the essentiality of other genes. When studying *M. pneumoniae* and *Mycoplasma agalactiae*, the genes involved in the production of an essential metabolite in a linear metabolic pathway were found to be essential (Montero-Blay et al., 2020). However, with two pathways that produce the same essential metabolite, the genes from both were classified as fitness genes and not essential, showing that both can be deleted but in reality, one of the two is necessary (Montero-Blay et al., 2020). The opposite of this is also seen where essential genes can be rendered nonessential in response to the deletion of a different gene in the genome. The issues with epistatic interactions and redundancies within the genome can be addressed by performing multiple rounds of Tn-Seq after genomic deletions but this is time consuming and labour intensive (Hutchison et al., 2016). Also, this does not take into account genes that do not act as part of larger networks.

To complement the identification of individual nonessential genes, a method combining Tn-Seq and Cre-LoxP named LoxTnSeq was developed to highlight large genomic regions that are nonessential (Shaw et al., 2020). By modifying the transposon to also carry a lox site, two lox sites can be randomly inserted into the genome and a deletion of random size is created by activating the Cre recombinase, which causes recombination between two lox sites orientated in the same direction. These deletion mutants can then be screened in different conditions and sequenced, same as Tn-Seq. LoxTnSeq was applied in *Mycoplasma pneumoniae* yielding a pool of mutants with 285 unique deletions ranging from 50 bp to 28 kb (21% of the total genome) (Shaw et al., 2020). Following deletion, a pitfall in previous methods is that the selection of the deletion mutant relies on screening for the loss of antibiotic resistance (Tsuge et al., 2007; Leprince et al., 2012). LoxTnSeq is designed to address this by forming an inactive lox72 site after deletion which cannot be acted on by Cre (Shaw et al., 2020). The constitutively expressed Cre recombinase is lethal in *M. pneuomiae* in the presence of active loxP sites, killing cells that do not have the deletion (Shaw et al., 2020).

Another approach to determining gene essentiality is CRISPR interference sequencing (CRISPRi-Seq), which employs a catalytically dead mutant of Cas9 that silences gene expression rather than causing a double stranded break (Figure 1) (Rousset et al., 2018; Liu et al., 2021a). By using a pool of over 90,000 sgRNAs that target random genomic loci within *E. coli*, 21% of the previously annotated essential genes were found to be nonessential. Initially, there were toxicity issues when dCas9 was combined with some specific PAM-proximal sequences in sgRNAs (Cui et al., 2018). After analysing the guides within the constructed sgRNA library, the pool was filtered down to ∼23,000 sgRNAs. Like Tn-Seq, after integration of the sgRNA library, silenced essential genes will be depleted and those with repression in nonessential regions will survive. Thus, following sequencing, the number of reads from the remaining cells can be compared to the initial pool to determine gene essentiality. Another similar study was run using around 60,000 guides within *E. coli* and despite having almost 3 times the number of guides in the library, both studies achieved similar results (Wang et al., 2018). Improving sgRNA design methods and minimizing the library size would ultimately enable more experiments to run concurrently and decrease the cost of DNA sequencing and synthesis. A comparison between using CRISPRi-Seq and TnSeq to determine gene essentiality can be found in Table 1. One advantage of this method over Tn-Seq is the ability to study duplicated regions in the genome. The gRNAs will target a specific region in the genome based on homology surrounding a PAM site, so multiple copies of the same gene region given identical sequences can be repressed at the same time, providing a more accurate representation of that gene repression. On the other hand, with TnSeq, the transposon will randomly integrate into one genomic region and is not sequence specific so if there are duplicate copies of the same gene, even if its essential, it may read as nonessential due to the complementation from the other copies. Additionally, the repression level can be somewhat regulated by modifying the gRNA target or sequence homology where transposons will insert and interrupt a gene without any level of control on the effect of such insertion. Finally, CRISPRi-Seq can have wider applications by targeting specific locations of genes by simply altering the gRNA pool where transposons integrate randomly with no specificity. This method has since been applied to create a library of gRNAs to target the core essential genome of 18 *E. coli* strains to compare gene essentiality within various genetic backgrounds (Rousset et al., 2021). Similar methods were also applied in *Streptococcus pneumoniae*, *Vibrio natriegens*, and *Synechocystis* sp. PCC 6803 (Lee et al., 2019; Yao et al., 2020; Liu et al., 2021a).

**TABLE 1:** Comparison between TnSeq and CRISPRiSeq methods to determine gene essentiality.

Characteristic	TnSeq	CRISPRiSeq
**Mutant Library Generation**	**Mutant library construction:** Conjugation of strain harboring suicide plasmid with transposon and transposase. Induce transposase activity, select, and pool resistant mutants	**gRNA library construction:** Random selection and synthesis of 10,000s of gRNAs, PCR amplification of synthesised guides, assembly into dCas9/guide expression plasmid, and transformation
		**Mutant library construction**
		Plasmid extraction from sgRNA library and electroporation into target *E. coli* strains
**Library Screening**	Growth of mutant library in desired conditions/media over multiple generations	Induction of dCas9 expression and growth of mutant library in desired conditions/media over multiple generations
**Sequencing Preparation**	Varies depending on the transposon used but for Mariner transposons; genome digestion to isolate transposon, blunting, and PCR with sequencing adaptors	PCR to amplify gRNAs
**Duplicated Gene Screening**	Only 1 transposon insertion per cell so cannot identify and screen those with duplicated genes	sgRNA can bind to multiple copies of the same region so can more effectively determine essentiality of those regions
**Inactivation control**	No control	Can modify the repression level by altering the sgRNA homology
**Genomic target**	Random integration^a^	Targeted integration by sgRNA sequence

a Depending on the transposon used (ie. mariner based transposons integrate into TA, dinucleotides but randomly across the genome).

#### 4.1.3 Essentiality determination by computational approaches

Computational programs and models have been developed to complement and fill in some gaps of time consuming and expensive experimental approaches of determining the essential genes. Using computational biology to assist with essential gene predictions is not something new. Comparative genomics, discussed above, has been used since 1996 and machine learning models to predict protein dispensability were developed in 2005 (Mushegian and Koonin, 1996; Chen and Xu, 2005). With technology advancements, these models and programs have recently become more sophisticated to address some challenges with computational approaches. One of the biggest challenges is that a minimal set of genes with a minimized metabolic network may not be viable in a cellular environment and/or not kinetically feasible. Thus, this is considered at all stages when developing a program to simulate essential gene predictions. The main features considered in computational models include expression level, sequence composition, evolutionary conservation, domain information, and network topology (Dong et al., 2018). These features have been reviewed in depth by Dong et al., 2018 but are briefly described below. First, the expression level of essential genes is typically higher than nonessential genes (Lloyd et al., 2015). This feature can be used to complement other essentiality prediction features, but not used as a method of assessment on its own due to the variability of this occurrence. Next, sequence composition can be analysed as there are differences between the amino acid sequences of essential and nonessential genes. This is a newer method of essentiality determination but is applicable in all sequenced genomes without the requirement of protein functional data (Zhang and Zhang, 1994; Sarangi et al., 2013; Ning et al., 2014). Evolutionary conservation is commonly used as a marker for essentiality and is based on the notion that genes that persist against negative selection through evolution are likely to be involved in essential functions within the cell (Jordan et al., 2002; Bergmiller et al., 2012). Like evolutionary conservation, domain information of proteins is the specific conservation of regions of proteins that perform similar functions. Also, essential genes encoding proteins will have various domains found infrequently in other proteins, making the substitution of these functions difficult (Chen et al., 2015; Peng et al., 2015). The first application of domain information is from 2011 and since then has been used in various studies, but not as commonly used as evolutionary conservation (Deng et al., 2011; Cheng et al., 2013; Lin et al., 2019). Finally, network topology refers to the way proteins interact within the cell with essential genes more frequently found in the center of complex protein-protein interaction networks (Yu et al., 2004; Wang et al., 2012). This also applies to metabolic, gene co-expression, and transcriptional networks (Acencio and Lemke, 2009; da Silva et al., 2008). For the most part, essentiality studies combine many of these features such as looking at the topologies of networks combined with gene expression profiles (Plaimas et al., 2008, 2010; Deng et al., 2011).

Based on both experimental data and computational models, online databases of the essential genes for various species have been created. First, the Database of Essential Genes (DEG) has lists of essential genes in 66 bacterial strains based on experimental data and this list is constantly being updated (Zhang et al., 2004; Luo et al., 2021). This database is the most widely used thanks to its practical tools such as homology searches using the embedded BLAST tool and since it contains essential genetic elements outside of just protein coding genes like non-coding RNAs, regulatory sequences, and essential promoters (Peng et al., 2017). An associated resource, database of predicted essential genes (pDEG), was constructed in 2011 in the same format as DEG to contain predicted essential genes for various *Mycoplasma* genomes but has not been updated since then to include other species or strains (Lin and Zhang, 2011). Two databases of essential genes for thousands of species have been more recently released, ePath and NetGenes (Kong et al., 2019; Senthamizhan et al., 2021). ePath, released in 2019, contains essential gene predictions for more than 4,000 bacteria (Kong et al., 2019). Its predictions are based on KEGG Ortholog (KO) annotations for biological functions, thus limiting this program to genes with KO numbers available. For example, a *Streptococcus* strain has 819 of its 2,270 genes annotated with KO so only those genes can be assessed for essentiality. This can be bypassed by using a built-in KEGG program, BlastKOALA to computationally assign KO numbers, but the obvious issue is that this is an estimation and not from experimental data (Kanehisa et al., 2016). The output for ePath includes essentiality scores based on experimental data (E-score) and the gene’s involvement in critical cellular processes (P-score) with prediction accuracies of 75–91% (Kong et al., 2019). NetGenes, released in 2021, has predictions for over 2,700 bacterial species and includes information on the essential genes like essentiality scores and feature vectors (Senthamizhan et al., 2021). The essentiality predictions rely on protein-protein interaction network-based features from the STRING database described in depth in an initial publication from 2018 (Azhagesan et al., 2018). This database provides a strong model for essential and nonessential gene classification but fails to capture fitness genes that are not essential but required for robust growth. So, while this data is important, this model is not yet constructed to be used independently to design a strong growing minimized genome cell. Other databases including OGEE (Online GEne Essentiality database), EGGS (Essential Genes on Genome Scale), CEG (database of essential gene clusters) are briefly described in Table 2.

**TABLE 2:** Essential gene databases and computational programs to predict essential genes and design genomic deletions.

Program	Function	URL	References
**DEG 15**	Database of essential genomic regions based on experimental data with embedded BLAST tools for homology searches	www.essentialgene.org/	Luo et al. (2021)
**pDEG**	Database of predicted essential genes within *Mycoplasma* strains	http://tubic.org/pdeg/	Lin and Zhang, (2011)
**NetGenes**	Database of predicted essential genes for more than 2,700 organisms based on protein-protein interaction network features	https://rbc-dsai-iitm.github.io/NetGenes/	Senthamizhan et al. (2021)
**ePath**	Database of predicted essential genes for more than 4,000 organisms based on previous experimental data and functional KEGG orthologs	https://www.pubapps.vcu.edu/epath/	Kong et al. (2019)
**OGEE**	Database of essential genes from experimental data for 91 bacterial strains with additional gene features like expression profiles, conservation, evolutionary origins etc.	http://ogeedb.embl.de	Chen et al. (2017)
**EGGS**	Database of essential genes from experimental data for 11 different species with visualization and analysis on a subsystem diagram	https://pubseed.theseed.org/FIG/eggs.cgi	Gerdes et al. (2006)
			Overbeek et al. (2005)
**CEG**	Database of essential genes based on data from DEG that are clustered based on function	http://cefg.uestc.cn/ceg	Liu et al. (2020a)
			Ye et al. (2013)
**CEG_Match**	Within the CEG database, bases the prediction of essential genes on function	http://cefg.uestc.cn/ceg	Liu et al. (2020a)
			Ye et al. (2013)
**ZCURVE 3.0**	Predicts genes from an unannotated genome and will also predict essential genes from that	http://guolab.whu.edu.cn/zcurve/	Hua et al. (2015)
**Geptop 2.0**	Predicts gene essentiality based on sequence conservation and orthology with comparing a fully sequenced organism to 37 other species	http://guolab.whu.edu.cn/geptop/	Wen et al. (2019)
**EGP**	Machine learning-based method applying sequence composition features to predict essential genes with the input of nucleotide sequence	http://cefg.uestc.edu.cn:9999/egp	Ning et al. (2014)
**Deeply Essential**	Deep neural network to identify essential genes in bacteria based on sequence features only	https://github.com/ucrbioinfo/DeeplyEssential	Hasan and Lonardi, (2020)
**DELEAT**	Prediction of essential genes based on 6 features not dependent on experimental or functional data with design of large genomic deletions to minimize the organism’s genome	https://github.com/jime-sg/deleat	Solana et al. (2021)
**MinGenome**	Minimal genome design with large deletion predictions using whole cell models using biological knowledge on gene locations and essentiality	https://github.com/maranasgroup/MinimalGenome	Wang and Maranas, (2018)
**GAMA**	Comprehensive and computationally intensive simulation of gene essentiality with gene deletions to construct a true minimal cell that cannot be run on a single computer	https://github.com/GriersonMarucciLab	Rees-Garbutt et al. (2020)
**Minesweeper**	Simultaneously assesses genomic regions that can be deleted, starting with larger deletion regions moving to individual genes	https://github.com/GriersonMarucciLab	Rees-Garbutt et al. (2020)

In addition to all the databases on essential genes, open-access programs have been created to run essential gene predictions, summarized in Table 2. Geptop predicts bacterial essential genes based on phylogeny, assessing evolutionary distance between species using composition vector method, and orthology, finding similar proteins across genomes using the reciprocal best hit method (Wei et al., 2013). This program, initially created in 2013, has recently been updated to include more essentiality data, increasing from 19 to 37 species, and to increase computation speed (Wen et al., 2019). Geptop 2.0 is simple to use with an interface to input DNA or protein sequences and receive the predicted essentiality with probabilities of genes or proteins but can only be used with fully sequenced organisms (Wen et al., 2019). CEG_Match, an extension on the CEG database, predicts essential genes based on function (Ye et al., 2013). More specifically, it matches the annotated gene names with the cluster gene names within the CEG database, avoiding issues with BLAST searches by eliminating the misclassification of genes with different sequences but similar functions (Ye et al., 2013; Peng et al., 2017). So here, the obvious limitation is that this prediction method only works with genes with known functions and names.

Machine learning is also an increasingly popular route of determining gene essentiality and has been reviewed extensively (Liu et al., 2020b, 2021b; Aromolaran et al., 2021). Briefly, machine learning is the ability of a computer system to ‘improve’ and ‘learn’ using inputted data to make predictions despite not being programmed to do so accurately (Aromolaran et al., 2021). So, data from model organisms on essential and nonessential genes are used to train a classifier that is then applied to predict gene essentiality in the same or a different organism. Many machine learning models have been created to analyze protein and genomic features which have been now applied to essential gene determination (Peng et al., 2017). These models are trained using sequence derived features and context-dependant features (Cheng et al., 2013; Ning et al., 2014). Sequence derived features include various factors like GC content, codon usage, protein length (more large and small proteins compared to medium sized proteins coded by essential genes), strand bias, and more (Lipman et al., 2002; Gong et al., 2008; Peng et al., 2017). Context-dependent features include those previously discussed like protein domain properties, protein-protein interaction networks, protein localization, and gene expression (Jansen et al., 2002; Seringhaus et al., 2006; Acencio and Lemke, 2009; Deng et al., 2011; Peng and Gao, 2014). Machine learning methods to predict essential genes are improved compared to homology mapping because they can use more features when constructing the prediction model (Deng et al., 2011; Lu et al., 2014). A machine learning-based method for essentiality predictions called Essential Gene Prediction (EGP) is freely accessible and only requires nucleotide sequence input (Ning et al., 2014). EGP uses amino acid, codon, and nucleotide usage as well as codon features independently to build the prediction model. This is done using training datasets from 16 genomes with known essential genes (Ning et al., 2014). It has been successfully used to identify essential genes in many organisms including *Salmonella typhimurium* and *E. coli* (Plaimas et al., 2010; Deng et al., 2012). But the selection of features and combinations may influence the performance of prediction and there is no clear method of selecting suitable features for differing organisms (Mobegi et al., 2017). Also, it has lower accuracy than some newer methods due to the limited reference species and parameters used for estimation (Peng et al., 2017). In general, a limitation of machine learning models is the inability to predict quasi-essential genes. Furthermore, there is a lack of complete and correct data from experimental and computational studies which impacts the accuracy of essential gene prediction in machine learning models.

Deep learning, a subset of machine learning, has networks that can use unlabeled or unstructured data to learn unsupervised. DeeplyEssential is a deep neural network that utilizes this learning model to predict essential genes by using only sequence information (Hasan and Lonardi, 2020). This model was able to achieve higher sensitivity and precision compared to clustered and down-sampled datasets used previously (Liu et al., 2017; Hasan and Lonardi, 2020). DeeplyEssential has countless applications since it only requires the genome sequence of the organism compared to other models that require topological or structural data which may not be available. Another deep learning model for essentiality predictions was developed taking a different approach using a framework that automatically learns biological features without the requirement of prior information (Zeng et al., 2021). This network uses information on gene expression, subcellular localization, and protein-protein interaction networks to learn topological features (Zeng et al., 2021). A major drawback to deep learning models is the high computational costs when training the network (Aromolaran et al., 2021). And when using them specifically for predicting essential genes, they require training with big data to outperform traditional machine learning algorithms and there is high complexity with tuning the parameters in deep learning models (Aromolaran et al., 2021). The biggest advantage of this approach is the ability to train algorithms with data from model organisms and use that to predict essential genes in poorly annotated organisms. But, as mentioned previously, it is unable to predict conditionally essential and quasi-essential genes. So in general, computational methods for essential gene prediction are great tools to supplement the experimental methods but require further improvements to be used as an independent tool.

### 4.2 Strategies for constructing reduced-genome bacterial cells

**FIGURE 2:**
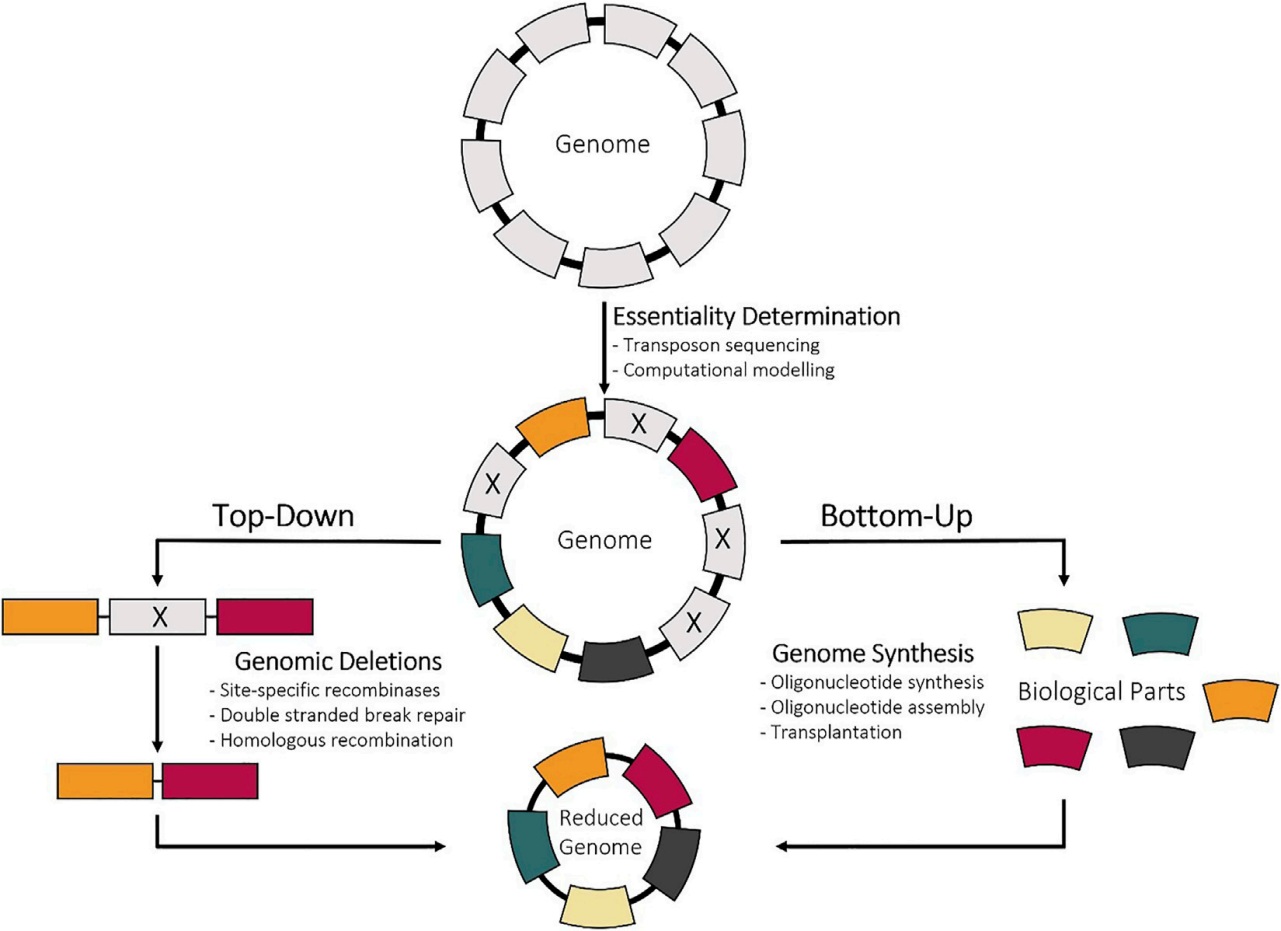
Experimental methods of determining gene essentiality. TnSeq inactivates a random gene by randomly inserting a transposon from a vector. LoxTnSeq deletes a random genomic region by inserting two transposons containing LoxP sites and activating recombination by Cre. CRISPRi-Seq inactivates a random gene by expressing a random gRNA and dCas9 that will bind and repress expression. For all three, the mutants will be pooled and subjected to various conditions followed by sequencing to identify remaining transposon location or guide RNA.

**FIGURE 3:**
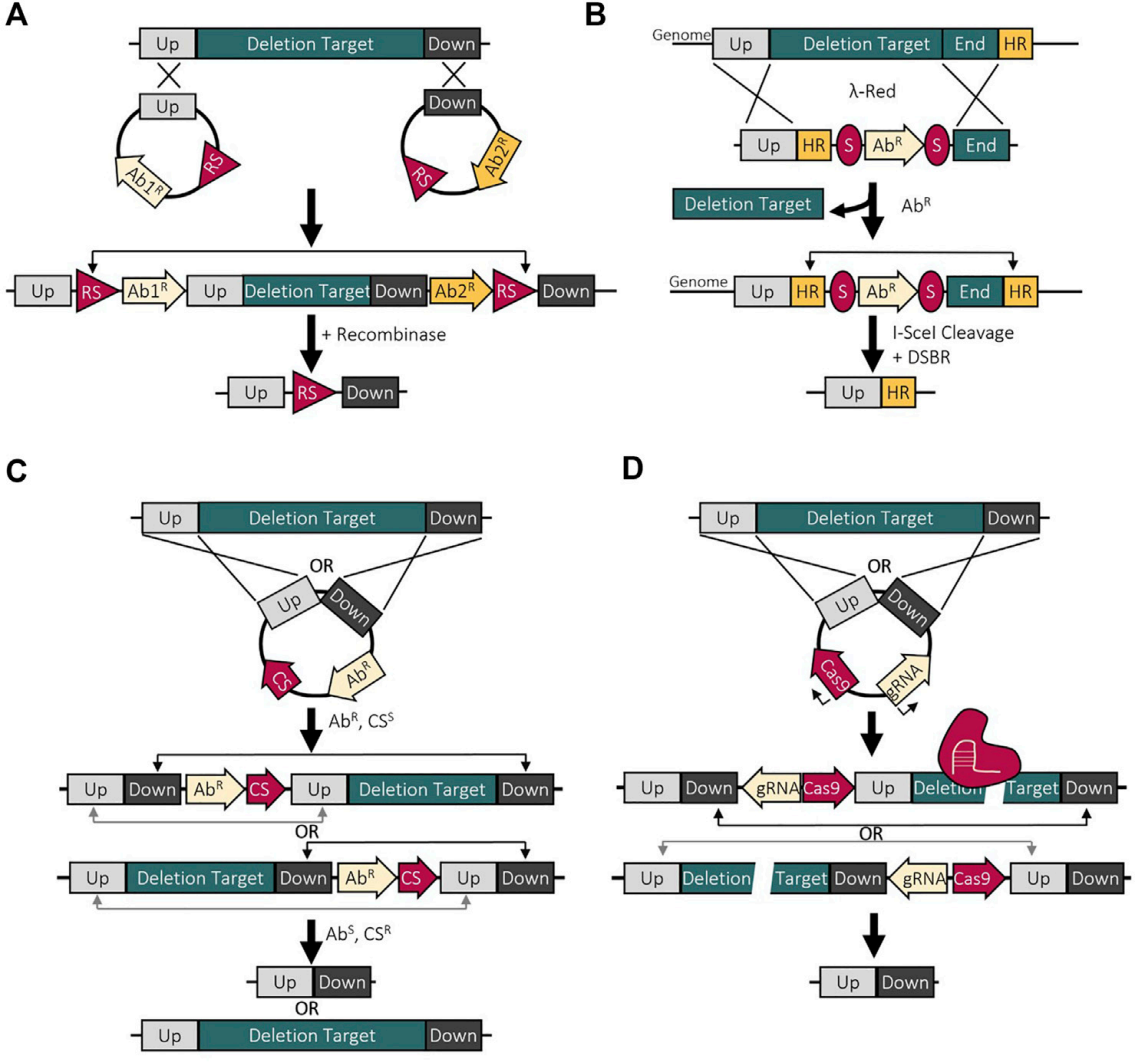
Deletion methods used to make large genomic reductions. **(A)** Site-specific recombination deletion method using two recombination sites (RS) which are acted on by a recombinase to result in recombination and deletion of the target. **(B)** λ red recombineering with I-SceI uses a linear DNA fragment with I-SceI cut sites (S) to cause a double-stranded break with recombination between the homologous regions (HR) to repair, resulting in a deletion. **(C)** Homologous recombination mediated deletions using a counterselectable marker (CS) to select for the second recombination event resulting in the deletion or return of wild-type. **(D)** CRISPR-based deletions employing Cas9 to cause a double-stranded break, forcing the cell to repair using homologous recombination, resulting in the deletion.

Once the essential genes are identified by combining the computational and experimental methods outlined above, the next step is to reduce genome. This can be done either using bottom-up methods, chemical synthesis of the minimized genome, or top-down methods, deletion of nonessential genes from the genome (Figure 2). Bottom-up approaches have been enabled with advancements in sequencing, gene synthesis, and assembly technologies. This method also offers the unique opportunity to not only create a cell with a smaller genome, but to also restructure the genome for biotechnological applications. However, top-down construction approaches are more popular and have been utilized more since they do not require completed genetic information (Sung et al., 2016). Also, the wide variety of deletion methods, many of which tailored to specific species, enables the use of this approach in almost any bacterial strain. Since the focus of this review is the minimization of genomes, methods that are used to make large deletions or multiple sequential deletions will be discussed, and are summarized in Figure 3. Prior to making deletions, what deletions to make and the order to make them needs to be determined.

#### 4.2.1 Computation design of genome reductions

As more is learned about bacterial genomes, the process of deciding which genes to remove and how to remove those genes becomes increasingly complex. Similar to using computational tools to predict gene essentiality, a few programs have been developed to assist in the deletion selection and genome design. The design of a tailored cell factory that produces key biomolecules or is a chassis for downstream applications is hindered by the segmented nature of our knowledge. Even with the knowledge and tools of how to build synthetic genomes bottom-up, very few have been constructed and reported due to the difficulty of designing such genomes (Hutchison et al., 2016; Richardson et al., 2017; Fredens et al., 2019). This stems from little understanding of genome design principles due to the inordinate complexity of target organisms. There is also a lack of ability to analyze and evaluate genomic designs and an overwhelming number of possible genome configurations even for bacteria with small genomes (Matteau et al., 2020). Even taking the smallest organisms, like *M. genetalium* with a total of 525 genes, there are 2^525^ possible genome-scale designs, making it impossible to assess all these designs *in vivo*. Thus, computational whole-cell models (WCMs) have been developed to simulate the dynamics of a cell but are currently limited mainly to model organisms due to lack of genomic annotation (Karr et al., 2012; Chalkley et al., 2019; Münzner et al., 2019; Norsigian et al., 2020). These algorithms can model the effect of large genomic deletions on growth rate and metabolism prior to experimental testing and assist in designing genomes with a minimal set of genes in an optimal configuration.

Three algorithms currently exist to run these genome reduction simulations; MinGenome, Minesweeper, and GAMA (Guess/Add/Mate Algorithm) (Table 2) (Wang and Maranas, 2018; Rees-Garbutt et al., 2020). MinGenome highlights long regions of nonessential genes by incorporating biological knowledge like gene location and essentiality with a genome metabolic model (Wang and Maranas, 2018). It also assesses large genomic regions that could be deleted even including regions that contain one or two essential genes that could be reintroduced after. This algorithm was applied to the *E. coli* MG1655 genome and showed similar results to experimental studies as well as alternative deletion combinations that have not been attempted in cells yet. Minesweeper takes a slightly different approach in that it assesses all genes that can be removed from the genome simultaneously, resulting in multiple different genome constructs since the order of deletions matters (Rees-Garbutt et al., 2020). It starts by deleting genes in groups then moves to deleting individual genes. This algorithm is based on gene knockout simulations to determine essential and nonessential genes. GAMA, developed at the same time as Minesweeper, first considers nonessential gene deletions in the guess/add stage before adding in essential genes in the final mate stage (Rees-Garbutt et al., 2020). In the guess phase, all the nonessential genes from the input (which is a preprocessing stage to determine nonessential genes) are broken into four sets of genes which are used to make around 400 subsets where combinations of genes are deleted and determined if cell division can occur with those deletions. These viable sets are taken to the add phase, where deletion sets from previous groups are combined into a larger deletion set. About 3,000 of these combined deletion sets are tested and the ones that produce a viable cell are ranked, taking 50 of the smallest genomes to the mate phase. In this phase, two of the 50 minimized genomes are mated with random knockouts and knock-ins from a pool of the protein coding genes. 1,000 simulations per mating is performed and the updated strain is passed back into the pool for another round, which is continued for 100 generations. The simulations with GAMA are very computationally heavy, using between 400 and 3,000 CPUs, taking over 2 months to process on a standard supercomputer to generate minimal genome size reductions. Thus, a previously developed genome design suite was utilized to implement GAMA (Chalkley et al., 2019). Whole cell models were used to simulate 10 sets of minimal genes from literature of *M. genetalium in silico* and it was found that those cells could not divide and replicate based on this algorithm (Rees-Garbutt et al., 2021). Each of these gene sets had deleted essential genes, even those that were compiled from TnSeq knockout studies. After reintroducing up to 26 genes considered to be essential and ‘low essential’, these gene sets could divide again *in silico*. This highlights a disconnect between available data on minimized gene sets and strains developed with various *in vivo* studies. While the new strains developed from this modeling have not been tested experimentally, this shows the potential of such models to improve existing strains and create new reduced genome strains.

Finally, another program called DELEAT (DELetion design by Essentiality Analysis Tool) has been created which combines *in silico* gene essentiality predictions and automatic large-scale deletion design across all bacterial genomes (Solana et al., 2021). To estimate gene essentiality, genes are assigned an essentiality score from 0 to 1 based on 6 gene features that don’t rely on functional annotation or experimental data. The deletion design is based on two parameters, the minimum desired deletion length (L) and the essentiality score (E). Following manual revision of the deletions, the program will provide a summary with various factors including deletion size, number of deletions, and deletion order. An additional feature will design primers to use to make the deletion constructs based on megapriming. This was used to highlight 35 deletions that could be made in *Bartonella quintana* to reduce the genome by 29% (Solana et al., 2021). While this program has yet to be fully tested, it provides promising insights into rapidly designing reduced cells. This is the first program that enables deletion design, from what to delete all the way to the deletion order and primer design.

#### 4.2.2 Site-specific recombination deletion methods

Moving to making genomic deletions, site-specific recombinases are often used due to their high deletion efficiency and functionality across multiple strains. Two methods that are frequently used and function similarly are Flp/FRT and Cre/loxP which causing recombination between *FRT* or *loxP* sites by the Flp and Cre recombinases respectively. When these sites are oriented in the same direction, recombination will result in the excision of the region between the two sites where recombination between two sites facing opposite direction results in an inversion. So, two vectors containing *FRT* of *loxP* sites can be introduced into the genome via homologous recombination, one upstream and one downstream of the deletion target (Figure 3A). After introduction of the recombinase, the deletion target is excised from the genome leaving behind one *FRT* or *loxP* site. This method has been used to make very large deletions at high efficiency but with the left-over recombination site, sequential deletions cannot be made without removing the leftover site (Komatsu et al., 2010). Improvements to Cre/loxP have been made to bypass this issue by utilizing *loxP* mutations to render the left-over *loxP* site non-functional. This allows new functional *loxP* sites to be reintroduced into the strain for subsequent deletions. This has been applied in *Bacillus pumilus* by using mutated *lox71* and *lox66* sites which result in a double mutant *lox72* site after recombination which has very low affinity for Cre (Guan et al., 2017). This does allow for multiple deletions but there will still be many *lox72* sites throughout the genome which is not ideal when constructing a reduced-genome chassis strain. Another downfall with site-specific recombination deletion methods is the tedious deletion mutant selection process. Each vector inserted with the recombination sites would have a selectable antibiotic resistance gene marker to select for the double integration. The cells would then have to be screened for sensitivity to the selected antibiotics to distinguish between those with the deletion and those with an inversion. This can be avoided in some species since the constitutive activity of Cre on active *loxP* sites can be toxic, like in *M. pneumoniae*, but this is not true for all bacteria. There are also modifications to the Flp/FRT system to include a counter-selectable marker to enable selection of deletion mutants (Ishikawa and Hori, 2013).

#### 4.2.3 Homologous recombination deletion methods

To combat some of the issues highlighted previously, alternative methods are used including markerless, homologous recombination-mediated, counter-selectable deletions. This involves the insertion of homologous regions up and downstream of the deletion target into a suicide vector that contains a selectable antibiotic resistance marker and a counter-selectable marker (Figure 3B). Common counter-selectable marker systems include sucrose (*sacB*), fusaric acid (*tetAR*), streptomycin (*rpsL*), and 5-fluorouracil (*upp*) (Reyrat et al., 1998; Fabret et al., 2002; Kang et al., 2002; Kristich et al., 2005; Goh et al., 2009; Keller et al., 2009). The first recombination event (insertion of the vector into the genome) can be selected for on the antibiotic plate and the second recombination event (excision from the genome) can be counter-selected on one of the above substances. If the vector is still present, the counter-selectable marker will result in cell death. This method is widely used as it is a ‘scarless’ deletion method and leaves no trace in the genome. Deficits of this method include the lower deletion efficiency compared to site-specific deletion methods. In the second recombinant deletion event, there is a 50% chance to excise only the plasmid from the genome, leaving the deletion target intact. In practice, this 50% efficiency of deletion is rarely obtained with a more realistic efficiency ranging from 10–40% (Graf and Altenbuchner, 2011). Also, this efficiency drops quickly as the size of deletion increases. For example, deletions of 26 and 64 kb achieved efficiencies of 40 and 20% respectively (Graf and Altenbuchner, 2011). It is possible to make very large deletions shown by the deletion of 1.4 Mbp in *Streptomyces,* but extensive screening was required to isolate cells with the correct deletion (Komatsu et al., 2010). This same deletion made with Cre-LoxP achieved 100% deletion efficiency (Komatsu et al., 2010). So, while this is a scarless deletion method with counterselection, screening is required to isolate wildtype and deletion mutants which is very time consuming when making multiple deletions.

To avoid the time-consuming screening, two routes have been taken, making concurrent deletions and combining into one strain after and the development of novel deletion methods. A deletion method combining λ red recombineering and I-SceI mediated double-stranded break repair was applied in *Pseudomonas* to make sequential genomic deletions (Chen et al., 2016). λ red recombineering employs three enzymes to catalyze homologous recombination of double stranded linear DNA with chromosomal DNA, avoiding the need to do any assembly steps outside of the cell (Yu et al., 2000). So, the linear substrate DNA contains an antibiotic resistance gene flanked by two I-SceI recognition sites (S) with 500 base pair homologous regions up- and down-stream of the deletion target (Figure 3C). After electroporation, I-SceI recombination removes the antibiotic resistance cassette, and the cell repairs the double stranded break using RecA-mediated homologous recombination (Chen et al., 2016). Similar methods have been applied in other species such as *Corynebacterium glutamicum* and *E. coli* (Vernyik et al., 2020; Wu et al., 2020). In *E. coli*, an approach using a Tn5 transposon containing I-SceI made successive, scarless deletions in the genome in random locations (Vernyik et al., 2020). This resulted in strains with up to a 2.5% genomic reduction with improved growth characteristics including biomass yield. Interestingly, of the 60 genomes sequenced, deletions were observed in the same 12 regions (Vernyik et al., 2020). The second method used to avoid time consuming sequential deletions employs the use of phage transduction to combine independent genomic deletions into one strain (Pósfai et al., 2006; Umenhoffer et al., 2017; Saragliadis et al., 2018). This allows for multiple deletions to be made at the same time, lowering the overall time required. This is also useful for transferring deletions made in one strain to a different strain and has been applied extensively in *E. coli* (Umenhoffer et al., 2017). This is limited by the host range of the phage but with the existence of multiple transducing phages, there are many strains that are compatible with this method of deletion combination (Kang et al., 2002; Lee et al., 2004; Saragliadis et al., 2018).

#### 4.2.4 CRISPR/Cas deletion methods

One of the most popular genome editing tools in members of the Eukarya domain are CRISPR-Cas systems. Despite being successful in some bacterial model strains, CRISPR technologies are not widely used in other bacteria in contrast to the increased use in Eukarya organisms (Dicarlo et al., 2013; Jiang et al., 2013; Mali et al., 2013). CRISPR-Cas systems are divided into two classes; class 1 consisting of multi subunit complexes and class 2 which are large, multi-domain proteins composed of one single unit (Makarova et al., 2020). Class 2 systems have been most used in bacteria, including Cas9, and Cas12, and typically involve the creation of double stranded breaks in DNA which signal repair through homologous recombination or non-homologous end joining. The Cas nuclease can be targeted to a specific location by gRNAs and will cleave complementary DNA that is flanked by a protospacer adjacent motif (PAM) (Gasiunas et al., 2012; Jinek et al., 2012). Often in bacteria, DNA cleavage and the overexpression of these large nucleases are lethal, limiting their use for making genomic deletions (Vento et al., 2019; Arroyo-Olarte et al., 2021).

With regards to making large-scale genomic reductions, CRISPR-Cas systems have been applied in two ways, to counterselect successful deletion mutants, and to make deletions. The lethality exhibited by the DNA breakage by a Cas nuclease can be used for counterselection. Cleavage is prevented when the PAM site is removed by a genomic deletion and those without the deletion will have a double stranded break, resulting in cell death (Oh and van Pijkeren, 2014; Banno et al., 2018; Penewit et al., 2018; Wirth et al., 2020). This is a useful method with high selection efficiencies with small deletions, but this efficiency drops significantly with larger deletions (Aparicio et al., 2018).

Additionally, there have been CRISPR-Cas systems developed to make genomic deletions successfully and rapidly in various bacterial species (Huang et al., 2015; So et al., 2017; Li K. et al., 2018; Zhang et al., 2019). These methods typically result in a deletion following DNA cleavage by a class 2 Cas nuclease and homologous recombination repair (Figure 3D). This employs either a heterologous recombinase or relies on endogenous homologous recombination machinery. Using native recombination machinery does simplify the overall process as it only needs one or two vectors harboring the CIRSPR-Cas elements and editing template, but this machinery may be lacking in some bacteria. For example, gene clusters in *Streptomyces coelicolor* of between 21 and 82 kb were deleted by using Cas9 with efficiencies between 38 and 100% (Huang et al., 2015). In addition, these gene clusters were deleted simultaneously with efficiencies between 29 and 54%, reducing the overall time to achieve a deletion by 3 times compared to other methods like Cre-LoxP. This involves the conjugation of *E. coli* harboring a plasmid with constitutive expression of Cas9 and gRNA as well as the up and downstream regions homologous to the deletion target. Following cleavage and two crossover events, only cells with the homologous recombination repair can survive. The plasmid can then be cured for another deletion round. A similar method using Cas9 was applied in *Bacillus subtilis* to achieve deletion efficiencies around 100% for a single gene and around 80% for a 38-kb region (So et al., 2017). Here, double stranded breaks were caused at the ends of the deletion region with homologous regions mediating repair in a two-plasmid system.

All these CRISR-Cas9-based editing methods that utilize recombineering either use linear DNA or circular DNA as the editing template, which each have their advantages and disadvantages (Jiang et al., 2013; Huang et al., 2015; Feng et al., 2018). A circular DNA has higher editing and recombination efficiencies since it can be copied along with plasmid replication and is not attacked by DNA exonucleases, but it is possible that the entire plasmid will be integrated into the genome, leading to high false positive rates (Huang et al., 2020). Linear DNA on the other hand does not have the issue with genomic integration so it has a higher positive rate, but it can be degraded by exonucleases. These challenges were confronted by bringing elements of both circular and linear DNA targets into one model. By adding the deletion template into a plasmid flanked by the target sequence, Cas9 cleavage can release this fragment from the plasmid. This protects it from degradation during the transformation process and achieves high positive rates of deletions by utilizing linear DNA editing templates. This method was used to delete 187 kb DNA regions from the *E. coli* genome with much higher editing efficiency and positive rates than other CRISPR-based λ-Red recombineering methods (Huang et al., 2020). This was taken a step further to make 12 sequential deletions totalling 370 Kb in *E. coli* (Huang et al., 2020).

Other barriers to using CRISPR-Cas9 deletion methods is the cytotoxicity observed in bacteria from over-expression of Cas9, even in its catalytically dead form (Li L. et al., 2018; Cho et al., 2018). Even in strains that can tolerate Cas9, editing efficiencies are lowered since there is a lower number of cells that survive (Xu et al., 2015; Li Q. et al., 2016; Song et al., 2017). This was improved by controlling the expression of Cas9 with inducible promoters, but leaky expression could still result in toxicity (Reisch and Prather, 2015; Wasels et al., 2017; Vento et al., 2019). Other alternatives include light-inducible systems, not yet attempted in bacteria, and the growth of cells at high temperatures to prevent Cas9 function, which is inactive above 42°C, but this requires bacteria that can tolerate this temperature (Polstein and Gersbach, 2015; Mougiakos et al., 2017; Nihongaki et al., 2018; Zhou et al., 2018). Using alternative Cas enzymes or mutated Cas9 variants is an option to circumvent this cytotoxicity such as the use of Cas9n that has a mutation that prevents double-stranded breaks, only allowing the enzyme to nick one strand of DNA (Jinek et al., 2012; Standage-Beier et al., 2015). Large deletions in a few different species have been achieved by using this method (Standage-Beier et al., 2015; Li K. et al., 2018). However, the Cas9n mutant has less efficient editing, especially with low expression of the enzyme (Song et al., 2017; Malzahn et al., 2019). So, this is a viable route to explore for a potentially more stable and universal CRISPR-based deletion method but needs to be enhanced first.

Another promising route is to explore the benefits and limitations of other Cas nucleases. A study has shown that the Cas12a nuclease could achieve efficient editing and transformation into *Corynebacterium glutamicum* while Cas9 and Cas9n could not (Jiang et al., 2017). Cas12a has been applied in *Clostridium difficile* to make a large 49 kb deletion and multiplex deletions, in *Streptomyces* to make single and double gene deletions with efficiencies between 75 and 95%, and in other species including *E. coli* and *Mycobacterium smegmatis* (Yan et al., 2017; Li L. et al., 2018; Hong et al., 2018). While these are a few successful examples, Cas12a is still a class 2 nuclease, meaning it is one large multi-subunit protein that can cause cellular toxicity when overexpressed.

More recent efforts have been using class 1 nucleases which are composed of multiple subunits (Xu et al., 2020). One use of these includes ‘built in’ genome editing using endogenously encoded CRISPR-Cas systems. This involves the identification of the CRISPR-Cas system that exists within the strain of interest and assembling a targeting plasmid that contains a minimal CRISPR array to target a specific genomic region. The deletion is mediated by cleavage followed by homology-directed DNA repair (Xu et al., 2020). Single deletions have been made successfully in various strains with high efficiencies including *C. saccharoperbutylacetonicum* (100%), *C. pasteurianium* (100%), *C. tyrobutyricum* (100%), *C. difficile* (30–100%), *L. crispatus* (100%), *P. aeruginosa* (50%), and *Z. mobilis* (100%) (Pyne et al., 2016; Zhang et al., 2018; Atmadjaja et al., 2019; Hidalgo-Cantabrana et al., 2019; Maikova et al., 2019; Xu et al., 2019; Zheng et al., 2019). Multiplex gene deletions have also been performed to simultaneously delete 2 genes in *C. tyrobutyricum* with 100% efficiency and 3 genes from *Z. mobilis* with 18.75% efficiency (Zhang et al., 2018; Zheng et al., 2019). Another example of a class 1 nuclease is the use of Cas3 to make non-specific deletions endogenously ranging from 7 to 424 kb in *Pseudomonas aeruginosa* with efficiencies reaching 100% as well as heterologously in *E. coli and Pseudomonas syringae* without a repair template (Csörgő et al., 2020). Though the targeting of Cas3 was not specific, it has great potential to make deletions much larger than Cas9 with higher efficiencies to make significant genomic reductions. To modify the functionality of Cas3, the nuclease was mutated to remove its helicase activity, converting the enzyme into a nickase (nCas3) that can nick single stranded DNA (Hao et al., 2022). Thus, two crRNAs were required to simultaneously target two genomic loci to get double nicking. This method was applied in *Z. mobilis* to generate a single deletion of 9 kb with an efficiency of 93.75% and to simultaneously delete two regions with an efficiency of 75% (Hao et al., 2022). Some other improvements include the creation of class 1 CRISPR-Cas systems that can be used in heterologous hosts. A transferable system employing a typeI-F cascade was created and used to make deletions up to 21 kb in *Pseudomonas* spp. with improved efficiencies compared to Cas9 systems (Xu et al., 2021). This was also further modified to incorporate λ-red for use in strains with poor homologous recombination and cells with anti-CRISPRs (Xu et al., 2021). While all these class 1 CRISPR-Cas tools were not used to construct a reduced genome cell, they still show promise for developing a system to make genomic reductions in strains with and without native CRISPR systems on a large scale.

Most studies making genomic deletions using CRISPR-based methods only highlighted its use to make a few deletions in a single strain. There are few studies that highlight a strategy that can be used across multiple strains and few that compare different methods of making the same deletions. One example is a strategy employing a RecT recombinase and Cas12a in various *Corynebacterium glutamicum* strains but was not very applicable to large deletions due to low efficiencies (Jiang et al., 2017). Another is the use of the typeI-F cascade in various *Pseudomonas* strains but has also only been tested on smaller, single deletions (Xu et al., 2021). Thus, with CRISPR-based methods, there is not one prevailing method, deletion strategies need to be tailored to the strain, genome size, deletion location, and number of deletions to be made.

Overall, CRISPR-based deletion methods show promising results for both improving counterselection methods and deletions made using homologous recombination but there is plenty of research still lacking on improving the efficiency of this system in many bacterial genetic backgrounds. Without a deletion method applicable across multiple species and for multiple deletion targets, this technology is very limited compared to other more universal methods. Many class 2 nucleases including Cas9 and Cas 12a have also only been applied in various model organisms with high transformation efficiencies, so are not directly applicable to many species yet. Class 1 nucleases including Cas3 show significant promise in improving cell toxicity and the size of genomic deletions but have not been used extensively in reducing the size of genomes or in the making of multiple genomic deletions. Taken together, reducing cytotoxicity, increasing deletion efficiencies, continued exploration of alternative Cas nucleases, and testing larger genomic reductions need to be addressed to make these technologies more applicable to large scale deletions in a wide range of bacteria.

## 5 Applications of reduced-genome bacterial strains

### 5.1 Genomic deletions for investigative research

The earliest reports of genomic deletions were applied to investigate the effect of various genes within the cell by deleting them from the genome. This often involved deleting only one gene at a time from the genome. For example, numerous studies investigated the effects of the deletion of *recA* in various strains. In *Mycobacterium bovis* BCG, a *recA* deletion revealed that the cell was more susceptible to DNA-damaging agents (Sander et al., 2001). In *E. coli*, a *recA* deletion increased transformation efficiency and improved *in vivo* phage packaging (Kurnit, 1989). The deletion of genes can also make some unexpected discoveries. In *Streptococcus pneumoniae*, the deletion of a zinc uptake lipoprotein, *adcAII*, revealed an unpredicted relationship with capsule thickness (Durmort et al., 2020). The capsule is the main virulence factor, but mechanisms involved in the regulation of its thickness is not well understood. Partial deletion of *adcAII* resulted in increased capsule thickness, making it hypervirulent and more resistant to neutrophil attack in mouse models (Durmort et al., 2020). Another discovery of novel functions to a previously annotated gene region was made in *Sinorhizobium meliloti* when removing almost half of its genes (diCenzo et al., 2016; Milunovic et al., 2014)*.* The genome of *S. meliloti* is divided into three components, a circular chromosome of 3.7 Mb, a chromid of 1.7 Mb, and a megaplasmid of 1.3 Mb. In this reduction, the chromid and megaplasmid were removed, moving the essential genes the chromid carries to the chromosome which uncovered four unexpected essential toxin/antitoxin genes, showing the first report that two of them even function as a toxin/antitoxin system (Milunovic et al., 2014).

Furthermore, deletion studies are also performed to investigate the role of bacterial genes in human pathogenesis. Again in *S. pneuomiae*, the deletion of a few genes residing in the Entner-Doudoroff pathway resulted in increased virulence and mortality in chinchilla models of otitis media (ear infection) (Hu et al., 2019). Overall, this study identified the role of various metabolic genes in virulence and pathogenicity including glucose dehydrogenase, the Entner-Doudoroff pathway, and ketogluconate degradation genes (Hu et al., 2019). These few examples of how genomic deletions can be utilized to investigate the role of various genes in a vast array of processes highlights their importance in both genomic and functional discoveries.

### 5.2 Genomic reductions to improve biomolecule production

Since bacteria are widely used in industrial processes to produce a variety of biomolecules, strains that are robust, able to survive in strenuous conditions, and have the natural ability to produce specific products are shortlisted for use at the industrial scale. These include strains of *Streptomyces sp*., *Pseudomonas sp*., *B. subtilis*, and *E. coli* (Kolisnychenko et al., 2002; Commichau et al., 2013; Belda et al., 2016; Calero and Nikel, 2019). By further modifying these organisms to synthesize products more efficiently, their applicability expands, and production costs are lowered. Techniques to improve biomolecule production include increasing precursor supply, enhancing flux through specific biosynthetic pathways, and reducing formation of by-products from alternative pathways (Gao et al., 2010). Genomic reductions can be targeted to these three areas such as making large genomic deletions to remove nonessential energy and resource consuming functions or through targeted deletions of competing pathways. Some biomolecules of interest and strains with genomic reductions to improve their production are highlighted below and summarized in Table 3.

**TABLE 3:** Previous genome reduced bacterial strains.

Strain	Deletion	Deletion size	Deletion method	Characteristics (relative to parental strain)	References
***Bacillus amyloliquefaciens***					
**GR167**	Genomic islands, extracellular polysaccharide biosynthesis genes, prophages	167 Kb (4.18%)	HR with *upp* CS	Faster growth, higher transformation efficiency, increased heterologous gene expression	Zhang et al. (2020)
***Bacillus subtilis***					
**∆6**	Prophages, *pks* operon	323 Kb (7.7%)	HR with no CS	Comparable growth rate	Westers et al. (2003)
**MG1M**	Prophages, antibiotic production genes	991 Kb (24%)	HR	Reduced growth rate, unstable recombinant protein production	Ara et al. (2007)
**MGB874**	74 regions including prophages, secondary metabolite producing genes, etc	873.5 Kb (20.7%)	HR with *upp* CS	Increase in cellulase (1.7-fold) and protease (2.5-fold) production	Morimoto et al. (2008)
**BSK814G2**	Prophages, antibiotic production operons and other nonessential regions	814 Kb (20%)	HR with *upp* CS	Decreased growth characteristics but 4.4-fold higher guanosine production	Li et al. (2016b)
**BSK756T3**	Prophages, antibiotic production operons and other nonessential regions	756 Kb (18.6%)	HR with *upp* CS	Decreased growth characteristics but 5.2-fold higher thymidine production	Li et al. (2016b)
**PG10**	Many genes including those for sporulation, motility, secondary metabolism, prophages, secreted proteases, etc.	1.46 Mb (36%)	HR with *manP* CS	Decreased growth rate, lower resource utilization for information processing, improved production of ‘difficult proteins’ that cannot be produced in other *Bacillus subtilis* strains	Reuß et al. (2017)
					Suárez et al. (2019)
***Corynebacterium glutamicum***					
**MB001**	3 Prophages	204.7 Kb (6%)	HR with *SacB* CS	Improved growth under stress conditions, increased transformation efficiency, 30% increase in heterologous protein production	Baumgart et al. (2013)
C1*	Non-essential genes including prophages, unknown genesetc.	440 Kb (13.4%)	HR with *sacB* CS	Robust against stresses, improved growth stability, similar growth rates	Baumgart et al. (2018)
**CR101**	All prophages and IS elements	249.4 Kb (7.6%)	HR with *sacB* CS	Similar growth rate and transformation efficiency to MB001	Linder et al. (2021)
***Escherichia coli***					
**MDS42**	Insertion sequences	663.3 Kb (14.3%)	λ-Red HR with I-SceI + P1 transduction	Improved electroporation efficiency, similar growth rates	Pósfai et al. (2006)
**∆16**	Various deletions across the *E. coli* genome	1.38 Mb (29.7%)	λ-Red HR with *sacB* and *rpsL* CS + P1 transduction	Slower growth and abnormal cell morphology	Hashimoto et al. (2005)
**MGF-01**	Various nonessential gene regions	1.03 Mb (22%)	λ-Red HR + P1 transduction	1.5-fold higher cell density and 2x threonine production from an introduced gene cassette	Mizoguchi et al. (2007)
**MS56**	IS Elements, K-islands, flagella genes, LPS synthesis genes	1.1 Mb (23%)	λ-Red HR with I-SceI + *sacB* CS	1.6-fold faster growth and improved genomic stability	Park et al. (2014)
***Lactococcus lactis***					
**9K-4**	Prophages, integrases, and transposases	71 Kb (2.83%)	Cre-LoxP	Faster growth rate, increased biomass yield, improved heterologous gene expression 3-4-fold	Zhu et al. (2017)
**N8-8**	Prophages and genomic islands	176 Kb (6.86%)	Cre-LoxP	Shortened generation time by 17%, similar nisin yield	Qiao et al. (2022)
***Magnetospirillum gryphiswaldense***					
**∆TZ-17**	Prophages, transposases, nitrogen fixation genes, *pks* operon	227 Kb (5.5%)	HR with *galK* CS	Comparable growth rate and magnetosome biosynthesis with improved genomic stability	Zwiener et al. (2021)
***Mycoplasma mycoides***					
**JCVI-syn3A**	All nonessential or quasi essential genes	669 Kb (55.2%)	Chemical synthesis	Improved growth rates compared to JCVI-syn3.0	Breuer et al. (2019)
***Pseudomonas alloputida***					
**KTU-13**	Genomic islands	254.5 Kb (4.1%)	HR with *sacB* CS	45-fold increase in transformation efficiency, 9.4-fold increase in heterologous protein expression, 39% increase in PHA production	Liang et al. (2020)
**EM383**	Flagellar biosynthesis genes, prophages, transposases, recombinases	265.8 Kb (4.3%)	HR with ISce-I	Improved growth rate, heterologous protein expression, plasmid stability, stress resistance, and more	Lieder et al. (2015)
					Martínez-García et al. (2014)
***Pseudomonas mendocina***					
**NKU421**	Genomic island, prophages, hypothetical protein clusters	418 Kb (7.7%)	HR with *upp* CS	Increased ATP/ADP ratio by 11x, Improved mcl-PHA and alginate oligosaccharide production by 114.8 and 27.8% respectively	Fan et al. (2020)
***Pseudomonas taiwanesis***					
**VBL120**	Megaplasmid, prophages, flagellar biosynthesis genes, and biofilm genes	640 Kb (10.7%)	I-SceI HR with CS	Increased growth rates and biomass yield, improved production of chemicals including phenol	Wynands et al. (2019)
***Sinorhizobium meliloti***					
**Rm1021**	2 megaplasmids containing nonessential genes including toxin/antitoxin systems	3.1 Mb (46%)	Flp/FRT	Identification of 4 toxin/antitoxin pairs that are essential	Milunovic et al. (2014)
***Streptomyces albus***					
J1074 (Del14)	15 biosynthetic secondary metabolite gene clusters	500 Kb (7.3%)	HR of mutant BAC library and *phi*C31 integrase	Comparable growth rates and improved heterologous gene expression of 7 products by 2–2.4 fold	Myronovski et al. (2018)
***Streptomyces avermitilis***					
**SUKA17**	Biosynthetic genes, prophages, transposases	1.67 Mb (18.5%)	Cre/LoxP	Increased streptomycin (4-fold) and cephamycin C (2-fold) production	Komatsu et al. (2010)
***Streptomyces chattanoogensis***					
**L321**	Biosynthetic clusters including the natamycin biosynthetic cluster	700 Kb (7.7%)	Cre/LoxP	Increased ATP and NADPH availability, higher transformation efficiency, improved heterologous gene expression, and increased genetic stability	Bu et al. (2019)

One area of interest is increasing the production of polyhydroxyalkanoates (PHAs). PHAs are biopolymers naturally synthesized by a variety of bacteria as a stored carbon source in limited nutrient environments with high availability of carbon sources (Khanna and Srivastava, 2005). They are a viable option for the replacement of environmentally harmful, petroleum-based plastics but adoption is hindered by the high production costs (Khanna and Srivastava, 2005; Mozejko-Ciesielska et al., 2019). Thus, genome reduction has been investigated as a strategy to improve PHA production. One strain of high interest due to its metabolic versatility and robustness, *Pseudomonas alloputida* KT2440, has been a target of such reductions (Mozejko-Ciesielska et al., 2019). Intuitively, the deletion of PHA depolymerase, encoded by *phaZ*, results in an increase of PHA yield by 38% compared to its parental strain (Poblete-Castro et al., 2014). Expanding on this, another study constructed a strain with the *phaZ* deletion and a deletion in two enzymes in a competing metabolic pathway, *fadB* and *fadA*, which resulted in a PHA yield increase of 13% when using an alternative carbon source, lignocellulosic biomass (Salvachúa et al., 2020). Next, the deletion of genomic islands accounting for approximately 4% of the genome also improved cell dry weight by 26.4% and PHA yield by 39.32% (Liang et al., 2020). Genomic reductions in other PHA producing strains have also shown strong improvements in production. By reducing the *Pseudomonas mendocina* genome by 7.7% with 14 sequential deletions, creating NKU421, the ATP/ADP ratio was improved by a factor of 11 and PHA production was improved by 114.8% compared to the parental strain (Fan et al., 2020). Another key organism that is of high interest to engineer improved biomolecule production is *Bacillus subtilis. B. subtilis* is widely used to produce enzymes and other chemicals at the industrial scale (Li Y. et al., 2016). Deletions totalling 814 kb in *B. subtilis* 168 found that transformation efficiency and growth rates were slightly decreased but when this strain was engineered to produce guanosine and thymidine by overexpressing some genes, accumulation increased 4.4- and 5.2-fold respectively (Li Y. et al., 2016). So, although some growth characteristics were negatively impacted, biomolecule production was improved significantly. This was observed in another minimized *B. subtilis* strain PG10 with a 36% genome reduction (Reuß et al., 2017). Despite a decrease in growth rate, this strain was able to produce ‘difficult proteins’ that could not be produced by other *B. subtilis* strains like staphylococcal antigens (Reuß et al., 2017; Suárez et al., 2019). Genome minimization has also been performed in non-model organisms to produce various biomolecules. For example, *Magnetospirillum gryphiswaldense* is a key organism in magnetosome biosynthesis and production (Zwiener et al., 2021). Magnetosomes have biological functions as magnetic sensors and have strong biomedical and biotechnological applications as magnetic nanoparticles in magnetic imaging, carriers for magnetic drug targeting and hyperthermia applications. Since large scale production is limiting its applicability, the genome of *M. gryphiswaldense* has been reduced by 5.5% to simplify its metabolism and work towards a chassis for magnetosome production (Zwiener et al., 2021). This strain displayed similar growth rates and magnetosome biosynthesis as the parental strain but had increased genetic stability and resilience (Zwiener et al., 2021). Overall, by reducing the genome size, producing biomolecules can be improved by freeing up more resources. This is often as a result of improved growth characteristics but there are cases where biomolecule production is increased, and other factors are worsened.

### 5.3 Genomic reductions to improve growth characteristics

Many of the deletions made above were made to improve growth characteristics and in turn increased biomolecule production. These factors include genomic stability, plasmid maintenance, growth rate, heterologous gene expression, and more. Research is also contributing to the creation of optimized chassis strains for any downstream application. These chassis are engineered for more rapid growth, improved stability, and improved heterologous gene expression. These strains can then be further engineered for uses such as producing a specific product otherwise unable to be produced by that organism or to utilize alternative carbon sources.

Looking at the prime example of *E. coli*, numerous studies have been conducted to improve its physiological characteristics. The genome of *E. coli* K12 MG1655, a common lab strain, was reduced by 14.3% by deleting insertion sequences resulting in improved electroporation efficiency and growth rates similar to the parental strain (Pósfai et al., 2006). In the same strain, a later study reduced the genome by 23% through the deletion of insertion sequences (ISs), K-islands, flagella genes, and some LPS synthesis genes (Park et al., 2014). These deletions resulted in 1.6-fold faster growth in minimal media and improved genome stability from the elimination of IS transposition (Park et al., 2014).

Moving away from *E*. *coli*, *Bacillus subtilis* 168 was reduced, creating MBG74, by deleting 874 kb of nonessential genomic regions, or 20% of the genome (Morimoto et al., 2008). This strain showed significant improvements in heterologous gene expression with increased yields of cellulase (1.7-fold) and protease (2.5-fold) (Morimoto et al., 2008). In *Bacillus amyloquefaciens*, 4.18% of the genome was deleted, making strain GR167, improving transformation efficiency, growth rates, and heterologous gene expression (Zhang et al., 2020). These growth characteristics highlight this strain as a suitable chassis for further genetic modification and industrial applications. As a proof of concept, GR167 was engineered to produce surfactin with two deletions and introduction of a stronger promoter in front of the native surfactant producing gene. These modifications, making strain GR167IDS, resulted in a 10.4-fold increase in surfactin compared to GR167 (Zhang et al., 2020). This highlights both the ease of manipulation of the reduced genome strain as well as the benefit of combining these reductions with other modifications to improve the expression of either native or foreign genes.

Strains that show promising growth characteristic improvements can be utilized as chassis for further downstream engineering. By combining these more stable and faster growing strains with either the integration of various biomolecule producing cassettes or by introducing more targeted deletions, they can be used as molecular production factories. For example, in *B. amyloliquefaciens*, the deletion of three peptidoglycan hydrolase genes resulted in an increased production of alpha-amylase by 48% and increased cell viability because of decreased cell lysis (Zhang J. et al., 2021). A future step to assess further improvements on alpha-amylase production could be to combine these three deletions into the minimized *M. amyloliquefaciens* GR167 strain. Or similarly, in a reduced genome *E. coli* strain, the deletion of *fadR*, *fabR*, and *iclR* has previously been shown to increase l-threonine production and could be introduced into a genome reduced strain for further optimization (Yang et al., 2019).

### 5.4 The consequences of minimal genomes

When reducing a genome, there is always the possibility of removing genes that may not be essential to survival or biomolecule production but essential to robust cell growth. This is highlighted by the minimal genome strains which contain only the genes needed for cell survival. The initial focus when constructing minimal genomes was in *E. coli* with the first strains constructed in 2002. The genome of *E. coli* K-12 MG1655 was reduced by 6.8% with 313.1 Kb deleted containing 287 open reading frames and 179 unknown genes, yielding strain *E. coli* CDΔ3456 with no improvement to growth characteristics (Yu et al., 2002). The construction of this strain uncovered that the deletion of certain pairs of genes that are individually nonessential resulted in cell death, termed synthetic lethal pairs. Another *E. coli* minimal genome strain published in 2005 had a deletion of 29.7% of the MG1655 genome resulting in strain *E. coli* Δ16 (Hashimoto et al., 2005). The deletion of these regions resulted in slower growth compared to the parental strain with almost two times the doubling time. The cells were also observed to have abnormal cell morphology and increased chromosome number per cell (Hashimoto et al., 2005). This was the first suggestion that minimal genomes may not be the end goal for having a chassis strain for downstream applications and that other genes unrelated to cell survival should be included in the final strain.

One of the most notable examples of a minimal genome is the *Mycoplasma mycoides* strain JCVI-syn3A (Breuer et al., 2019). After using whole genome chemical synthesis combined with assembly and cloning in yeast, *M. mycoides* synthetic genome was transplanted into *Mycoplasma capricolum*, making it the first cell that is controlled by a synthetic genome, JCVI-syn1.0 (Gibson et al., 2010; Sleator, 2010). The genome of this strain was further reduced to a total of 531 Kb and 473 genes, just under 50% the size of the parental strain (Hutchison et al., 2016). Named JCVI-syn3.0, this strain was an autonomously replicating cell with the smallest genome recorded (Hutchison et al., 2016). However, this strain displayed much slower growth rates, 2 to 3 times less than that of the parental *M. mycoides* strain and had some altered morphological traits. While this cell can survive and replicate, the elimination of all non-essential genes prevented it from being much use so the final revision to this strain occurred with the reintroduction of 20 genes to bring JCVI-syn3A to a total of 543 kb and 493 genes (Breuer et al., 2019). This included quasi-essential genes that are not required for survival but are required for robust growth with 149 of the protein coding genes having no known function (51). This emphasizes the fact that despite having the tools and technology to construct a synthetic minimal genome, there is still so much that is unknown about what set of functionalities are essential in enabling life and robust growth. A few studies following the strain construction looked at associating function to the unannotated proteins. By taking various approaches including sequence-based annotations, secondary structure matching, and multi-pipeline approaches, 66 proteins of unknown function in JCVI-syn3.0 were assigned function (Danchin and Fang, 2016; Hutchison et al., 2016; Yang and Tsui, 2018; Breuer et al., 2019). A more recent study annotated 50% of the proteins with unknown functions, 9 times more than existing UniProt annotations, by applying a novel pipeline that computationally predicted protein structure using map-based simulations followed by structural-based function annotation and protein-protein interaction predictions (Zhang C. et al., 2021).

There was initially a strong focus on creating minimal genome strains for use as chassis for downstream applications, but after seeing the effects on a cell, such as decreased growth rates and altered cell morphology, there was a shift towards only reducing a genome to the point where the organism can still have robust growth. Regardless, these minimal genomes are of immense value for research purposes and discovering the core set of genes required for bacterial life. The construction of minimal genomes has also elucidated many aspects of the genome such as the function and importance of specific genes or genomic interactions such as synthetic lethal pairs.

## 6 Discussion

Overall, genomic reductions are a promising avenue for uncovering gene functions, improving the production of valuable biomolecules and for creating chassis strains that can serve as model organisms on which to build applications. A combination of experimental and computational methods is likely to be most powerful for determining gene essentiality. For making defined deletions, many methods exist, and many are tailored and designed for specific species and strains With CRISPR becoming a popular tool in Eukaryal organisms, it is interesting to see it lagging in terms of applicability in bacteria, likely because powerful methods for genome manipulation have existed in bacteria for many years. Despite this, more recent research has been designing and implementing CRISPR-based methods for making deletions and with continued progress towards using alternative Cas enzymes, minimizing the toxicity of the class 2 nucleases, and improving deletion efficiencies, these efforts could result in improved approaches for large-scale deletion.

Since the ideation of minimal genomes, the goal with genomic reductions has shifted from making the smallest genome possible in a surviving cell to reducing the genome to make a cell that functions well and is not crippled. This is especially highlighted by the many studies that showed reduced growth characteristics in cells with extreme reductions. So, while minimal cells are valuable from a perspective of gaining knowledge on cellular functions and the essentials for survival, a reduced genome cell that has improved growth characteristics can be used for many applications and is the goal. One of the biggest driving forces behind the construction of genome-reduced hosts is the increasing demand for improved production of economically important bio-metabolites from stable, robust, and reliable strains. With an increasing number of reduced genome strains and more studies focused on improving methods of identifying essential genes and making deletions both experimentally and computationally, the area of large-scale genomic reductions is overcoming many challenges. Within the next few years, we can expect to see these reduced-genome alternatives serve as chassis for a broad range of applications.
